# Complete Genome Sequence of Stenotrophomonas maltophilia Strain CF13, Recovered from Sputum from an Australian Cystic Fibrosis Patient

**DOI:** 10.1128/MRA.00628-20

**Published:** 2020-08-06

**Authors:** Mohammad Hamidian, James Lazenby, Joyce To, Rebecca Hartstein, Jana Soares, Samantha McNamara, Cynthia B. Whitchurch

**Affiliations:** a The ithree institute, University of Technology Sydney, Ultimo, NSW, Australia; Loyola University Chicago

## Abstract

Stenotrophomonas maltophilia isolate CF13 is a multidrug-resistant isolate that was recovered in Sydney, Australia, in 2011, from a sputum sample from an individual with cystic fibrosis. The genome sequence of CF13 was completed using long- and short-read technologies.

## ANNOUNCEMENT

Stenotrophomonas maltophilia is as an important opportunistic pathogen associated with antibiotic-resistant respiratory infections globally (1–3). A collection of putative Pseudomonas aeruginosa isolates were isolated from sputum samples from individuals with cystic fibrosis at NSW Health Pathology (Prince of Wales Hospital, Randwick, NSW, Australia) in 2011 as described previously (4). One multidrug-resistant isolate (CF13) was selected for sequence analysis and was found in this study to be S. maltophilia. Antibiotic sensitivity testing was performed by standard broth microdilution (5, 6). The MICs for CF13 were as follows: 4 mg/liter (gentamicin), 32 mg/liter (amikacin), 128 mg/liter (meropenem), 2 mg/liter (polymyxin B), 2 mg/liter (colistin), >128 mg/liter (aztreonam), 256 mg/liter (ceftazidime), 64 mg/liter (ticarcillin-clavulanic acid), and 128 mg/liter (fosfomycin).

Whole-cell genomic DNA of CF13 was isolated using the DNeasy microbial kit (Qiagen), from cells grown overnight at 37°C in LB inoculated from a frozen stock derived from a single colony. Illumina HiSeq and MinION sequencing was performed at the ithree Core Sequencing Facility, University of Technology Sydney (Ultimo, Australia). Library construction for Illumina MiSeq sequencing was performed following the adapted Nextera Flex library preparation kit process Hackflex, as described previously (7). The library for MinION sequencing was prepared using the rapid barcoding sequencing kit (SQK-RBK004) and was sequenced using a FLO-MIN106D flow cell (identification number FAL30280) according to the manufacturer’s instructions (Oxford Nanopore Technologies, Inc., Oxford, UK). Illumina HiSeq sequencing generated 1,013,693 paired-end short reads with 50-fold coverage and an average length of 250 bp, and MinION sequencing produced a total of 23,907 reads with an *N_50_* value of 18.2 kbp and 30-fold coverage. FastQC (v.0.11.9) (https://www.bioinformatics.babraham.ac.uk/projects/fastqc) and Filtlong (v.0.2.0) (https://github.com/rrwick/Filtlong) were used to check the quality of Illumina and MinION reads, respectively. Filtlong filtered long reads by quality, length, and comparison to a reference genome (K279a, GenBank accession number AM743169), excluding reads that did not belong to S. maltophilia. Illumina and MinION reads were trimmed using the Trimmomatic (v.0.39) (8) and Porechop (v.0.2.3) (9) programs, respectively, using default parameters. The high-quality Illumina and MinION reads were assembled *de novo* using a hybrid assembly approach with the Unicycler program (v.0.4.7) (10), using default parameters, which resulted in a single chromosomal contig. Unicycler was used to rotate and open the chromosome at *dnaA* to determine that the genome was circular and complete, a method and program that were validated previously (10). The chromosome, consisting of 4,591,696 bases with a GC content of 66.46%, was annotated using the NCBI Prokaryotic Genome Annotation Pipeline (v.4.11) (11). The chromosome of CF13 encodes a total of 4,086 putative proteins, with 69 tRNAs and 6 rRNA regions. CF13 carries no plasmid.

**FIG 1:**
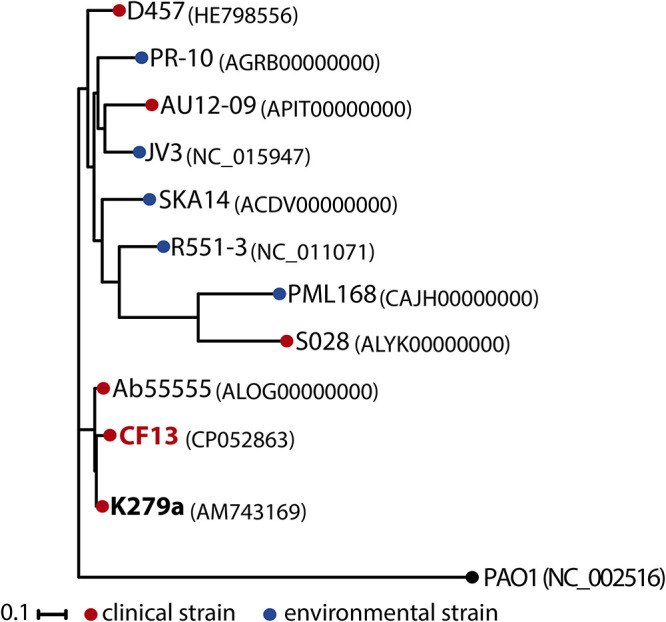
Core genome phylogenetic tree of S. maltophilia strains. Red and blue nodes indicate isolates recovered from clinical and environmental sources, respectively. The black node shows Pseudomonas aeruginosa PAO1, which was used as an outgroup. Numbers next to the strain names indicate GenBank accession numbers. Sequence reads were mapped to the reference strain K279a (17) using Snippy (v.4.6.0) (https://github.com/tseemann/snippy) to generate a whole-genome alignment. Variant sites were called using SAMtools (v.1.3.1.24) (9), and a maximum likelihood tree was inferred using RAxML (v.8) (21) with the generalized time-reversible gamma model of nucleotide substitution. Ten independent runs of RAxML with 1,000 bootstraps each gave nearly identical results; therefore, the first replicate was taken as a single representative result, with support values calculated from 1,000 bootstraps.

The complete genome sequence shows that CF13 belongs to a novel sequence type (ST) of S. maltophilia, namely, ST371 (with *atpD-5*, *gapA-62*, *guaA-289*, *mutM-158*, *nuoD-25*, *ppsA-116*, and *recA-103*). To determine the relationship of CF13 to other known strains (12–17), a phylogenetic tree was constructed. CF13 was found to be clustered with the reference strain K279a (Fig. 1). The population structure of S. maltophilia consists of 23 monophyletic lineages, with the largest lineage, Sm6, known as S. maltophilia *sensu stricto*, containing most strains characterized to date, including K279a (18). Hence, CF13 also belongs to Sm6.

CF13 contains the aminoglycoside resistance genes *aph(3')-II* and *aac(6')-I* and the *bla*_L1_ resistance gene, which encodes a broad-spectrum Ambler class B metalloenzyme. CF13 also carries the *oqxB* gene, which, in conjunction with *oqxA*, encodes a resistance-nodulation-division (RND)-type efflux pump that is involved in resistance to multiple antibiotics (19, 20). However, only a partial segment of *oqxA* was found in CF13.

### Data availability.

The complete genome sequence has been deposited in DDBJ/ENA/GenBank under the accession number CP052863. Illumina and MinION sequence reads have been deposited in the Sequence Read Archive (SRA) database under the accession numbers SRR9903515 and SRR11687566, respectively.
